# Chemosensory systems interact to shape relevant traits for bacterial plant pathogenesis

**DOI:** 10.1128/mbio.00871-24

**Published:** 2024-06-20

**Authors:** Martí Munar-Palmer, Saray Santamaría-Hernando, Janine Liedtke, Davi R. Ortega, Gema López-Torrejón, José Juan Rodríguez-Herva, Ariane Briegel, Emilia López-Solanilla

**Affiliations:** 1 Centro de Biotecnología y Genómica de Plantas, Universidad Politécnica de Madrid (UPM)–Instituto Nacional de Investigación y Tecnología Agraria y Alimentaria (INIA-CSIC), Madrid, Spain; 2 Institute of Biology, Leiden University, Leiden, The Netherlands; 3 Departamento de Biotecnología-Biología Vegetal, Escuela Técnica Superior de Ingeniería Agronómica, Alimentaria y de Biosistemas, Universidad Politécnica de Madrid (UPM), Madrid, Spain; The Ohio State University, Columbus, Ohio, USA

**Keywords:** chemoperception, *Pseudomonas syringae*, motility, biofilm, crosstalk, virulence

## Abstract

Chemosensory systems allow bacteria to respond and adapt to environmental conditions. Many bacteria contain more than one chemosensory system, but knowledge of their specific roles in regulating different functions remains scarce. Here, we address this issue by analyzing the function of the F6, F8, and alternative (non-motility) cellular functions (ACF) chemosensory systems of the model plant pathogen *Pseudomonas syringae* pv. tomato. In this work, we assign PsPto chemoreceptors to each chemosensory system, and we visualize for the first time the F6 and F8 chemosensory systems of PsPto using cryo-electron tomography. We confirm that chemotaxis and swimming motility are controlled by the F6 system, and we demonstrate how different components from the F8 and ACF systems also modulate swimming motility. We also determine how the kinase and response regulators from the F6 and F8 chemosensory systems do not work together in the regulation of biofilm, whereas both components from the ACF system contribute together to regulate these traits. Furthermore, we show how the F6, F8, and ACF kinases interact with the ACF response regulator WspR, supporting crosstalk among chemosensory systems. Finally, we reveal how all chemosensory systems play a role in regulating virulence.

**IMPORTANCE:**

Chemoperception through chemosensory systems is an essential feature for bacterial survival, as it allows bacterial interaction with its surrounding environment. In the case of plant pathogens, it is especially relevant to enter the host and achieve full virulence. Multiple chemosensory systems allow bacteria to display a wider plasticity in their response to external signals. Here, we perform a deep characterization of the F6, F8, and alternative (non-motility) cellular functions chemosensory systems in the model plant pathogen *Pseudomonas syringae* pv. tomato DC3000. These chemosensory systems regulate key virulence-related traits, like motility and biofilm formation. Furthermore, we unveil an unexpected crosstalk among these chemosensory systems at the level of the interaction between kinases and response regulators. This work shows novel results that contribute to the knowledge of chemosensory systems and their role in functions alternative to chemotaxis.

## INTRODUCTION

Signal transduction systems allow bacteria to perceive environmental stimuli and orchestrate an appropriate response to improve survival and growth. Among these systems are the chemosensory systems, specialized two-component signal transduction systems that arise from an evolutionary variation of the former to acquire an adaptative and amplified response (1). The best-known chemosensory system is the chemotaxis system, which allows the control of motility toward attractants or away from repellents by modulating the motility patterns (2). However, these systems appear to be involved not only in the control of flagellar function but also in the regulation of other cellular functions such as type IV pilus-based motility, biofilm formation, or the control of second messenger levels (3–5).

The core of a chemosensory pathway is formed by a complex composed of one or more chemoreceptors (CR) or methyl-accepting chemotaxis proteins, the CheA histidine kinase, and the CheW adaptor protein (6). In the canonical pathway, signal binding to the CR ligand-binding domain (LBD) creates a molecular stimulus that modulates CheA autophosphorylation, which in turn alters the transphosphorylation of the response regulator CheY. In the case of a chemotaxis pathway, phosphorylated CheY (CheY-P) binds to the flagellar motor, controlling the direction of flagellar rotation and ultimately bacterial motility. The adaptability of the system, allowing the response to stimuli over a broad concentration range, comprises the activity of the CheR methyltransferase and the CheB methylesterase, which, respectively, add and remove methyl groups from CRs modulating their affinity to the chemical stimulus. The pathway also features the CheZ phosphatase, which dephosphorylates CheY, thereby halting the signaling process (7).

The number of chemoreceptors present in a bacterium and their ligand-binding specificity are associated with its ability to perceive and respond to environmental changes. Diverse chemosensory systems have been found in different bacteria, showing variations in their components (8). Moreover, it is not unusual to find genes encoding different chemosensory transducer elements within a given bacterial genome, which suggests a versatile response capacity through different specialized pathways (9).

Chemosensory systems are classified into three main functional groups: those that evolve to control flagellar motility (F), type IV pilus-based motility (Tfp), and those involved in alternative (non-motility) cellular functions (ACF) like the control of levels of second messengers (10). The F group is the most diverse and contains 17 classes (F1–F17), while the other two groups contain a single system class.

Proteins of chemosensory systems (CRs and signal transducer elements) assemble into macromolecular complexes known as chemosensory arrays (11). Clustering enables chemosensory complexes to react collectively, thereby enhancing their sensitivity to fluctuations in environmental conditions (12, 13).

Different CRs present particular LBDs, which are the hallmark of their specificity. Genomic analysis of CR sequences predicts nearly 100 distinct LBD types (14, 15). However, the cytoplasmic signaling domain is less diverse and can be classified into seven classes based on the number and organization of the characteristic seven-residue heptads (H) present in this domain. This feature allows the classification of CRs based on the length of their signaling domain. The physical length of receptors determines whether they can be incorporated into the same chemosensory array (16, 17). This means that CRs with different ligand-binding specificities but with the same number of heptads can arrange together facilitating signal integration (18).

In bacteria with multiple chemosensory systems, the spatial segregation of arrays formed by each system, comprising different CRs, would be advantageous for preventing interference among different functions (19). However, crosstalk between different chemosensory systems has been reported (20–23). Therefore, it remains an open question if and how different chemosensory systems are structurally and functionally separated in a given species (11).

Phytopathogenic bacteria present a high number of CRs and often have multiple chemosensory systems. While 90% of phytopathogenic bacteria possess chemotaxis-related genes (14), such genes are only found in 47% of all bacteria (24). There is abundant experimental evidence that highlights the importance of chemotaxis for efficient plant colonization (25–36).

*Pseudomonas syringae* pv. tomato DC3000 (PsPto) is the causal agent of bacterial speck in tomatoes and is considered an aggressive pathogen once inside the plant (37, 38). However, it is a weak epiphyte compared to other *P. syringae* strains (39), suggesting that it needs to rapidly perceive and respond to environmental and plant signals to ensure entry into the plant apoplast (40, 41). Stomata are the main entry points of PsPto to the apoplast, and it has been shown that PsPto is able to actively move toward open stomata (42). Biofilm formation is also involved in adaptation to environmental stress such as that generated during the interaction between PsPto with plants (43). Pathogenicity has been shown to be associated with increased motility and decreased cell aggregation (44). Therefore, these traits must be tightly regulated to adapt to the host environment and ensure efficient plant colonization.

PsPto contains four main chemosensory systems in independent gene clusters: cluster I (F6), cluster II (F8), cluster III (ACF), and cluster IV (Tfp). The 49 CRs that interact with these systems are scattered across the genome. Previous results determined that cluster I is responsible for chemotaxis (33), as a defective *cheA2* mutant lost chemoattraction to several compounds. Moreover, this mutant also displayed an altered phenotype in the levels of the second messenger cyclic diguanosine monophosphate (c-di-GMP), suggesting crosstalk between chemosensory systems (33). In addition, it has been shown that a PsPto *cheA2* mutant is impaired in virulence in tomato plants (45).

Although previous studies on PsPto and *P. syringae* pv. tabaci have analyzed phenotypes associated with mutations in the F6 and F8 chemosensory systems in these bacteria (45, 46), the way the different chemotaxis pathways connect and interplay to facilitate adaptation to plant host has not yet been elucidated.

In this work, we propose an assignment of each of the 49 CRs to any of the main four chemosensory systems found in this bacterium. We show, for the first time, the visualization of the primary chemosensory arrays in this bacterium and analyze the function, crosstalk, and interaction among components of the main PsPto chemosensory systems (F6, F8, and ACF) in relation to pathogenicity traits.

## MATERIALS AND METHODS

### Bacterial strains, growth conditions, and plasmids

Bacteria and plasmids used in this study are listed in Table S1. PsPto derivatives were grown at 28°C in King’s B (KB) medium (47). *Escherichia coli* derivatives were grown at 37°C in lysogeny broth (LB) (48). When appropriate, antibiotics were added to the medium at the following concentrations: rifampicin 25 µg/mL, kanamycin 50 µg/mL, and ampicillin 100 µg/mL.

### Construction of deletion mutant strains and complementation strains

Generation of deletion mutants was carried out by double crossover and sucrose counter-selection using pK18mobsac (49). Complementation strains were obtained by expressing the corresponding gene in each mutant background using the broad-host-range plasmid pBBR1MCS-2 (50). The Δ*cheA2* was complemented using the pME6010::CheA2 plasmid (45). A detailed description can be found in Text S1.

### Prediction of chemoreceptor arrays’ height

To obtain the classification of chemosensory proteins and chemoreceptors, we used the MiST4.0 (51) database. We retrieved the protein sequence in fasta format and annotations of transmembrane regions. To build the atomic models from the sequences, we used ColabFold v1.5.2 (52) with default parameters. This version of ColabFold uses AlphaFold 2 (53) with MMseqs2 (54) in batch mode. The atomic structure was analyzed using VMD 1.9.4a57 (55) to measure the size of the receptor from the tip of the receptor to the amino acid from the transmembrane region closest to the cytoplasm.

### Cryo-electron tomography

For sample preparation, PsPto strains were grown overnight in KB liquid medium at 28°C. Cells were washed twice with M9 minimal media supplemented with 10 mM citrate and concentrated by centrifugation at 3,000 × *g* for 5 min. Protein A-treated 10 nm colloidal gold solution (Cell Microscopy Core, Utrecht University, Utrecht, The Netherlands) was added to the samples at a dilution of 1:20 and mixed gently before plunge-freezing. Subsequently, 3 µL of the sample was applied to a freshly glow-discharged, carbon-coated copper grid R2/2, 200 mesh grids (Quantifoil Micro Tools GmbH, Großlöbichau, Germany). Plunge-freezing was performed on an EM GP plunge freezer (Leica Microsystems, Wetzlar, Germany) in liquid ethane under the following conditions: pre-blotting time, 30 seconds; blotting time, 1 second; humidity, 85%; and chamber temperature, 20°C.

Data were acquired using a TITAN Krios 300 kV cryo-transmission electron microscope (Thermo Fisher Scientific, Hillsboro, OR, USA), and images were recorded with a K3 direct electron detector (Bioquantum, Gatan, Pleasanton, CA, USA) with a slit width of 20 eV. Images were acquired at a magnification of ×19,500, which corresponds to a pixel size of 4,411 Å. Tilt series were collected using SerialEM 4.1 (56) with a bidirectional tilt scheme from −60° to 60°, starting from 0° with 2° increments. The cumulative exposure was set to 157 e-/Å and the defocus to –6 µm. From each variant, 48–50 tilt series were collected, and three-dimensional reconstructions were generated using the IMOD software package (57, 58). The tomograms were screened for the presence of visible chemotaxis arrays. Only tomograms with chemotaxis arrays in side view were further processed.

### Measurement of chemotaxis arrays from cryo-tomograms

The measurement of the average array density profiles was calculated automatically using the “sideview-profile-average” script (59). Briefly, model points were generated in tomograms along the inner membrane and chemotaxis arrays. Based on these model points, the script calculates the average pixel value perpendicular to the model points in the same plane to create JSON files with the average pixel profile. The profiles were visualized in the ObservableHQ notebook to measure the distance between the density dips of the inner membrane and the CheA/CheW plane (Fig. S1).

### Capillary chemotaxis assays

Cultures grown overnight were diluted to an OD_600_ of 0.05 in KB medium and grown at 28°C with orbital shaking. At the early stationary phase of growth, cultures were centrifuged at 1,740 × *g* for 5 min, and the resulting pellet was washed twice with 10 mM HEPES (pH 7.0). Cells were resuspended in HEPES and adjusted to an OD_600_ of 0.25. Next, 230 µL of cells were placed into each well of a 96-well plate. One-microliter capillaries were filled with the compound to be tested, immersed into the bacterial suspension, and incubated for 30 min. Capillaries were removed from the bacterial suspension and rinsed with sterile water, and the content was expelled into 1 mL of nutrient broth (NB) medium (1 g yeast extract, 2 g beef extract, 5 g NaCl, and 5 g Bacto Peptone [per liter]). Serial dilutions were plated onto NB medium with the appropriate antibiotics, and the number of colony-forming units (CFU) was determined. In all cases, data were corrected by subtracting the number of cells that swam into buffer-containing capillaries.

### Swimming motility assays

PsPto strains were grown on KB agar plates at 28°C for 48 hours. Individual colonies were picked and inoculated into soft KB agar plates, M9 citrate (60) agar plates, or HRP (61) medium agar plates (0.3%, wt/vol), a medium that mimics plant apoplast. Soft agar plates were incubated at 28°C in a humid environment under dark conditions. The diameter of the swimming halo was measured at different time points.

### Attachment assays

Attachment assays were performed as described previously by Chakravarthy et al. (44), with a modified MG liquid medium, MGA medium (54 mM mannitol, 3.6 mM KH_2_PO_4_, 23 mM NaCl, 0.8 mM MgSO_4_, and 18 mM NH_4_Cl [pH 7.0]). A detailed description can be found in Text S1.

### Colony morphology and Congo red binding assays

Overnight cultures grown in KB liquid medium at 28°C were washed with KB medium and diluted to an OD_600_ of 0.05. Twenty microliters of diluted culture was inoculated either in KB agar plates (1.5%, wt/vol) supplemented with Congo red (40 µg/mL) and Coomassie brilliant blue (20 µg/mL) or M9 citrate agar plates (1.5%, wt/vol) supplemented with Congo red (40 µg/mL). Pictures were taken with ZEISS Axio Zoom.V16 stereoscope at different time points.

### Flocculation assays

PsPto strains were grown overnight at 28°C in KB medium and left statically at RT for at least 24 hours. Pictures were taken when flocculation was observed.

### Bacterial two-hybrid assays

Bacterial strains and plasmids were purchased from Euromedex (France). The reporter strain *E. coli* DHM10 (cya^-^) was co-transformed with two-hybrid plasmids. Clones were selected on LB agar plates supplemented with ampicillin (100 µg/mL) and kanamycin (50 µg/mL). Three clones of each co-transformation were inoculated in LB medium supplemented with kanamycin (50 µg/mL) and ampicillin (100 µg/mL) and grown at 37°C and 200 rpm for at least 4 hours. Two microliters of bacterial culture was spotted on LB plates supplemented with kanamycin (50 µg/mL), ampicillin (100 µg/mL), 0.5 mM IPTG, and X-gal (40 µg/mL). Plates were incubated at RT or 28°C until colonies turned blue. A detailed description can be found in Text S1.

### Virulence assays

PsPto strains were grown at 28°C for 24 hours on KB agar in the dark. Cells were resuspended in 10 mM MgCl_2_ and diluted to 10^8^ CFU/mL. For spray inoculation assays, 3-week-old tomato plants (*Solanum lycopersicum* cv. Moneymaker) were sprayed with a suspension containing 10^8^ CFU/mL, and Silwet L-77 was added at a final concentration of 0.02% (vol/vol). Plants were incubated in a growth chamber at 25°C and 60% RH with a daily light period of 12 hours. Six days post-inoculation (dpi), the leaf symptoms were recorded, and bacterial populations from three plants were measured by sampling five 1-cm-diameter leaf disks per plant. A detailed description can be found in Text S1.

For syringe-infiltration assays, cells were resuspended in 10 mM MgCl_2_ and diluted to 3 × 10^4^ CFU/mL. Tomato leaves were pierced with a needle on the adaxial side of the leaves, and 100 µL of bacterial suspension was syringe-infiltrated through the wound. Plants were incubated in a growth chamber at 25°C and 60% RH with a daily period of 12 hours. Three days after inoculation, the leaf symptoms were recorded, and bacterial populations were determined.

### Statistical analysis

All statistical analyses were performed using the GraphPad Prism software. When samples followed a normal distribution, a *t*-test or one-way analysis of variance (ANOVA) was performed. When ANOVA was used, multiple comparisons were performed using Fisher’s least significant difference *post hoc* test. When samples did not follow a normal distribution, a Mann-Whitney test or a Kruskal-Wallis test was used. Multiple comparisons were performed using Dunn’s test.

## RESULTS

### Assigning chemoreceptors to chemosensory systems in PsPto

**Fig 1:**
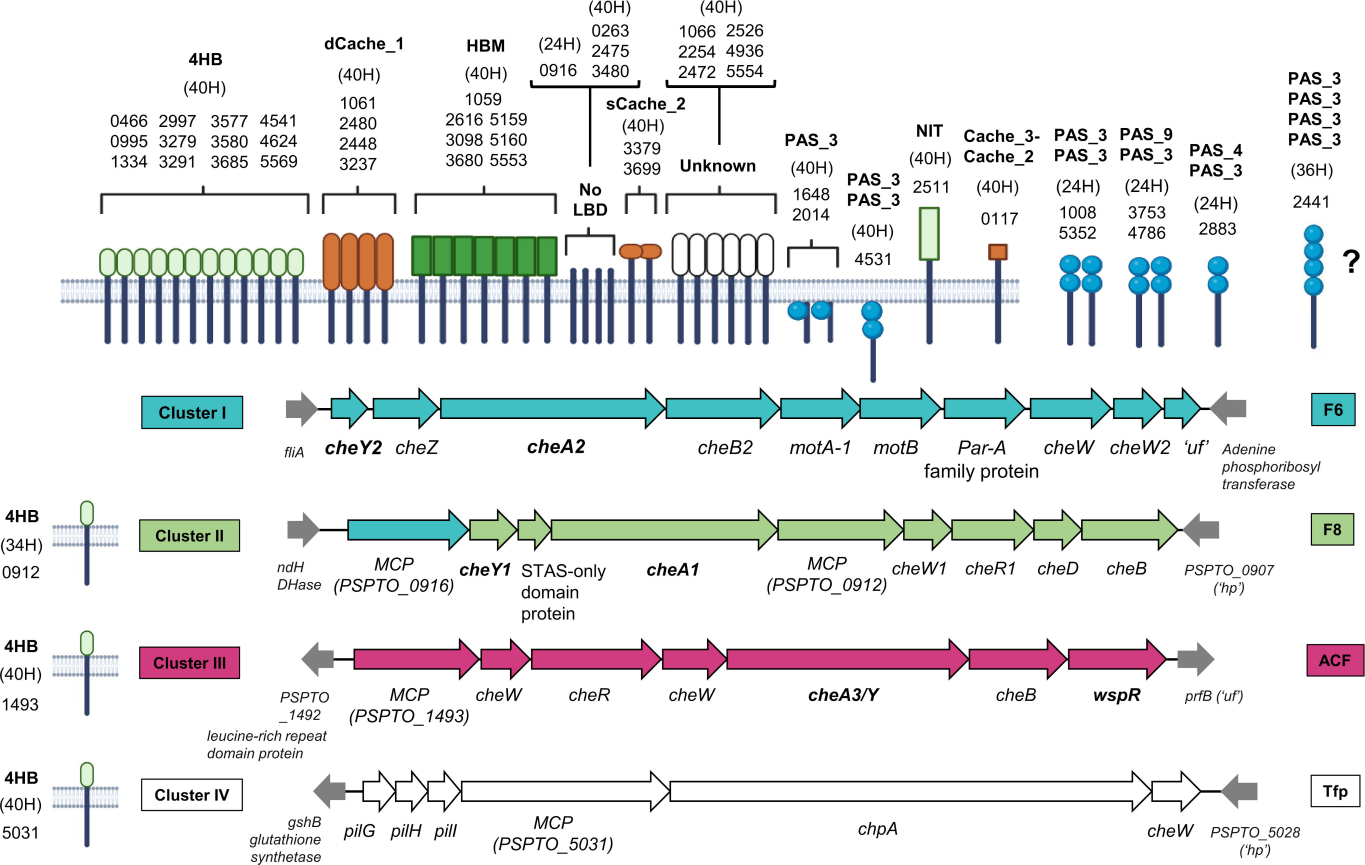
Chemoreceptors and chemosensory systems of *Pseudomonas syringae* pv. tomato DC3000. Chemoreceptors are grouped by LBD topology and chemosensory system association. Chemoreceptor class is indicated by the number of heptads (H). NCBI accession numbers (PSPTO_XXXX) are indicated below the class type. Nomenclature for the LBD types is the same as in Sanchis-López et al. (24). Genes mutated in this work are shown in boldface type.

Most PsPto chemotaxis genes are arranged in four gene clusters (clusters I–IV) called chemosensory systems. Additional chemotaxis genes are located across the genome with no particular organization. PsPto main clusters were initially numbered according to their homology with other *Pseudomonas* species (*Pseudomonas aeruginosa* PAO1 and *Pseudomonas putida* KT2440) (62) on the basis of their genomic organization. Genes in the clusters were annotated before the cluster numbering, and therefore the numbering of genes differs from the numbering of the chemosensory system they belong to (Fig. 1).

All chemotaxis and chemoreceptor genes from PsPto were retrieved using the MiST4.0 database (51). MiST4.0 associates chemotaxis genes with specific chemosensory categories depending on gene orthology, protein structure, and experimental evidence in related bacteria (63). MiST4.0 predicts that PsPto has 49 chemoreceptors (41 from the 40H class, 1 from the 36H class, 1 from the 34H class, and 6 from the 24H class). The four PsPto chemosensory systems are categorized as F6, F8, ACF, and Tfp (Fig. 1).

In bacteria with a single chemosensory system, the 24H receptor class has been assigned to work with F1, F3, F5, F6, and F7 systems (64). Since only the F6 system is present in PsPto, we assign all six 24H receptors to the F6 system (cluster I). The F6 system is known to also work with 40H receptors (64); therefore, we assign all 40H chemoreceptors to the F6 system, except for two chemoreceptors, PSPTO_1493 and PSPTO_5031. PSPTO_1493 exhibits synteny and shares 69.2% identity with the *wspA* gene of PAO1, with a genomic location in the ACF chemotaxis cluster. *PSPTO_5031* exhibits synteny and shares 77.4% identity with the *pilJ* gene of PAO1, encoded in the Tfp chemotaxis cluster. Therefore, we assign PSPTO_1493 and PSPTO_5031 to the ACF and Tfp systems, respectively (Fig. 1; Table S2). Moreover, experimental data are available on the homologous chemoreceptors of PAO1 showing their function in association with these classes of chemosensory systems (65, 66). PSPTO_0912 is the only receptor belonging to the 34H class, which is a minor class of chemoreceptors. PSPTO_0912 is encoded in the F8 gene cluster (cluster II), suggesting it functions in association with this system. Moreover, the assignment of 34H chemoreceptors to the F8 system has been previously proposed in other organisms (59, 67) (Fig. 1; Table S2). PSPTO_2441 is the only chemoreceptor belonging to the 36H class, which has been mostly assigned to the F1, F3, F7, and Tfp classes (64). From these systems, only Tfp is present in PsPto; therefore, we could assign PSPTO_2441 to the Tfp class (Fig. 1; Table S2). However, PSPTO_2441 is a cytoplasmic CR harboring four PAS domains. Most CRs with PAS domains have been assigned to the F6 system; therefore, a definitive assignment cannot be performed for this CR.

### Cryo-electron tomography reveals two distinct chemotaxis arrays in PsPto

Of all chemosensory systems found in PsPto, only the F6 type has been successfully imaged before in other bacteria using cryo-electron microscopy (59). To our knowledge, F8, ACF, and Tfp systems have not been previously imaged. In the case of ACF and Tfp systems, it is still unknown if they assemble clusters large enough to be visible with cryo-electron microscopy. Thus, we focused on building structural models for the F6 and F8 systems using two chemoreceptor sequences from classes 40H (PSPTO_2480) and 34H (PSPTO_0912). In the case of the 34H chemoreceptor, we measure 20 nm from the amino acid I215 at the base of the transmembrane region to the G380 at the tip of the receptor. In the case of the 40H chemoreceptor, we measure 23 nm from the amino acid M301 to the G488 at the tip of the chemoreceptor. Thus, we predict that the F8 system will have a height of 20 nm and the F6 system a height of 23 nm.

**Fig 2:**
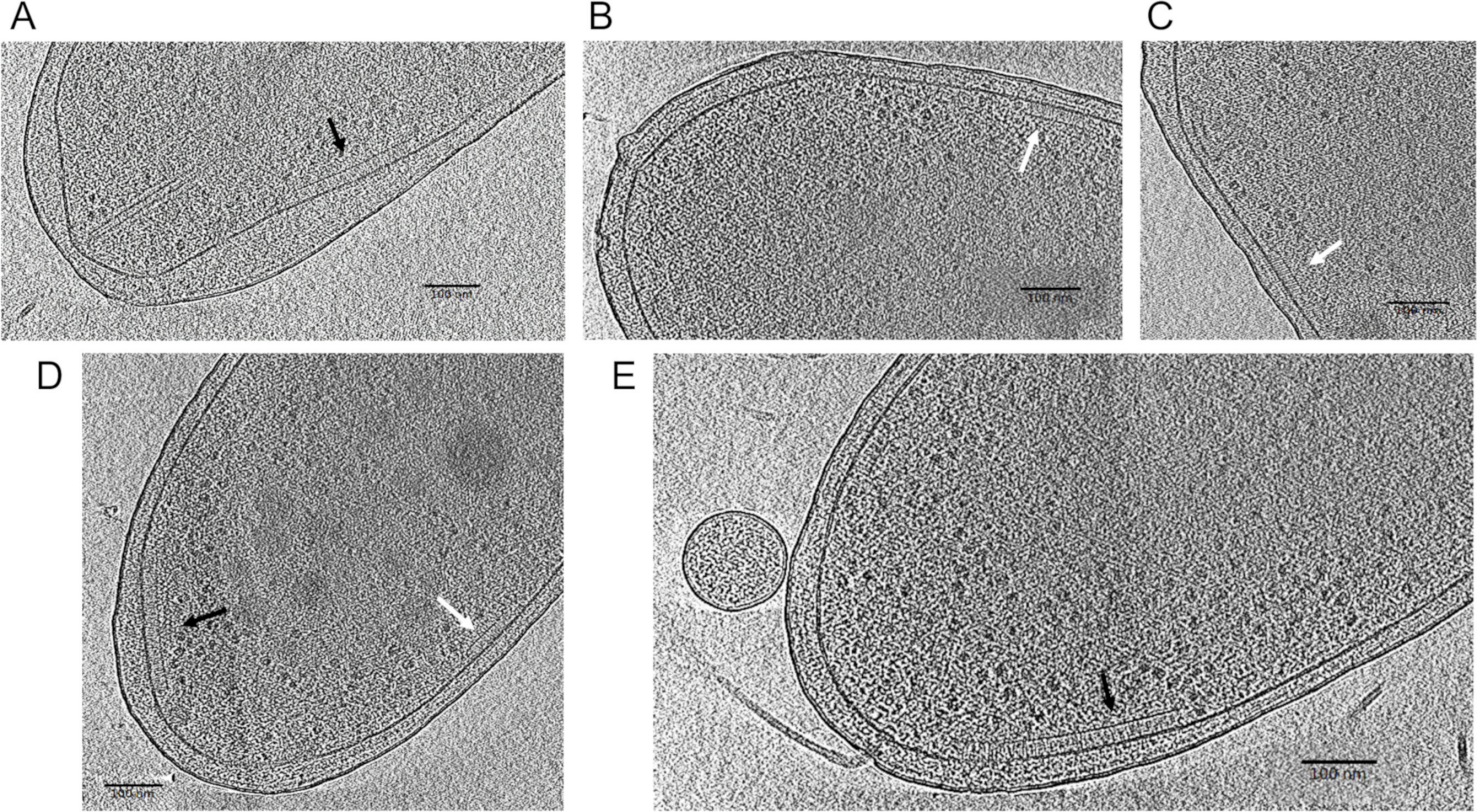
Cryo-electron microscopy of WT, Δ*cheA1*, and Δ*cheA2* cells from PsPto. Black arrows point to chemosensory arrays of type F6, and white arrows point to chemosensory arrays of type F8. (**A**) WT with F6 array. (**B**) WT with F8 array. (**C**) Δ*cheA2* with F8 array. (**D**) Δ*cheA2* with F6 and F8 arrays. (**E**) Δ*cheA1* with F6 array.

We used cryo-electron tomography to visualize and characterize the chemoreceptor arrays in PsPto (Fig. 2). We were able to visualize and identify two distinct arrays in wild-type (WT) cells measuring 20 and 23 nm in height. This is consistent with our predicted measurements, thus identifying the arrays as F8 and F6 systems, respectively. In *E. coli*, the absence of CheA kinase impedes the formation of the chemotaxis array. Since CheA1 is predicted to take part in the F8 array and CheA2 in the F6 array, we also imaged cells from Δ*cheA1* and Δ*cheA2* strains to confirm which kinase forms each array. Δ*cheA1* cells only showed F6 arrays, which indicates that CheA1 participates in the formation of F8 arrays. Surprisingly, Δ*cheA2* showed both F6 and F8 arrays (Fig. 2; Table S3).

### Chemotaxis is impaired in Δ*cheA2* but not in Δ*cheA1* and Δ*cheA3* PsPto mutant strains

**Fig 3:**
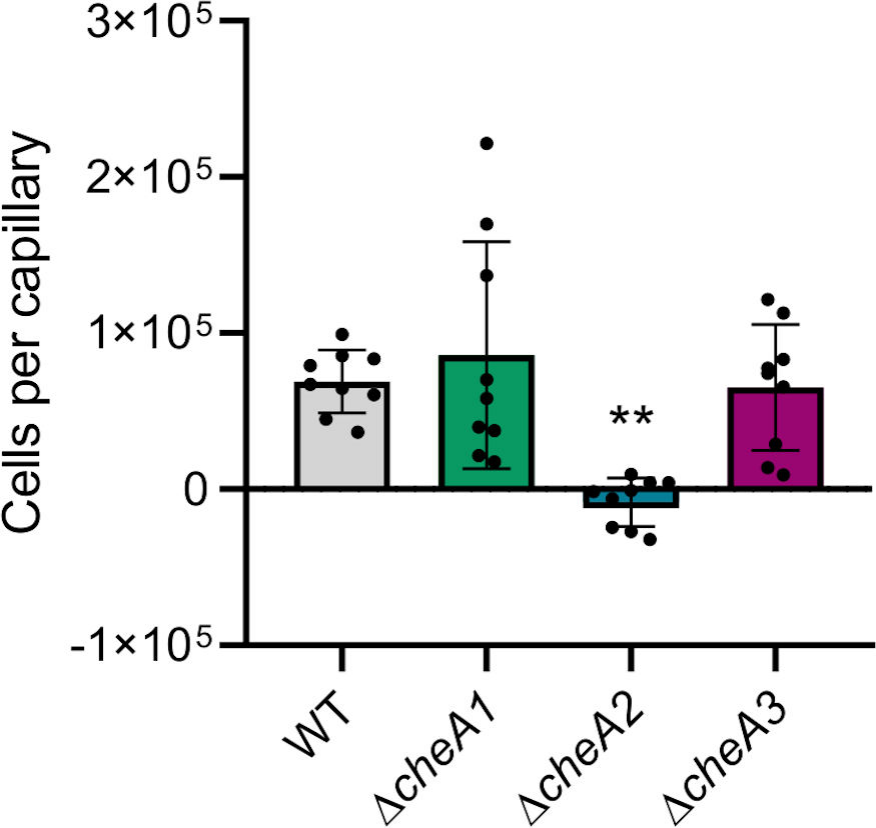
Chemotaxis capillary assays against 0.1 mM casamino acids. The data have been corrected with the number of cells that swam into buffer-containing capillaries. Bars represent the means of three independent experiments with three technical replicates each. Values that are significantly different are indicated by asterisks (**P  <  0.01).

To unveil the role of the different PsPto chemosensory systems in the control of flagellar motility, we performed capillary-based chemotaxis assays against casamino acids, which are strong chemoattractants for *Pseudomonas*. We tested this phenotype in the Δ*cheA1*, Δ*cheA2,* and Δ*cheA3* mutants altered in the kinase of each chemosensory system. Results showed that only the F6 system is involved in the control of chemotaxis in PsPto (Fig. 3). These results are in line with the previously reported involvement of this system in amino acid perception (33).

### Chemosensory systems regulate swimming motility

**Fig 4:**
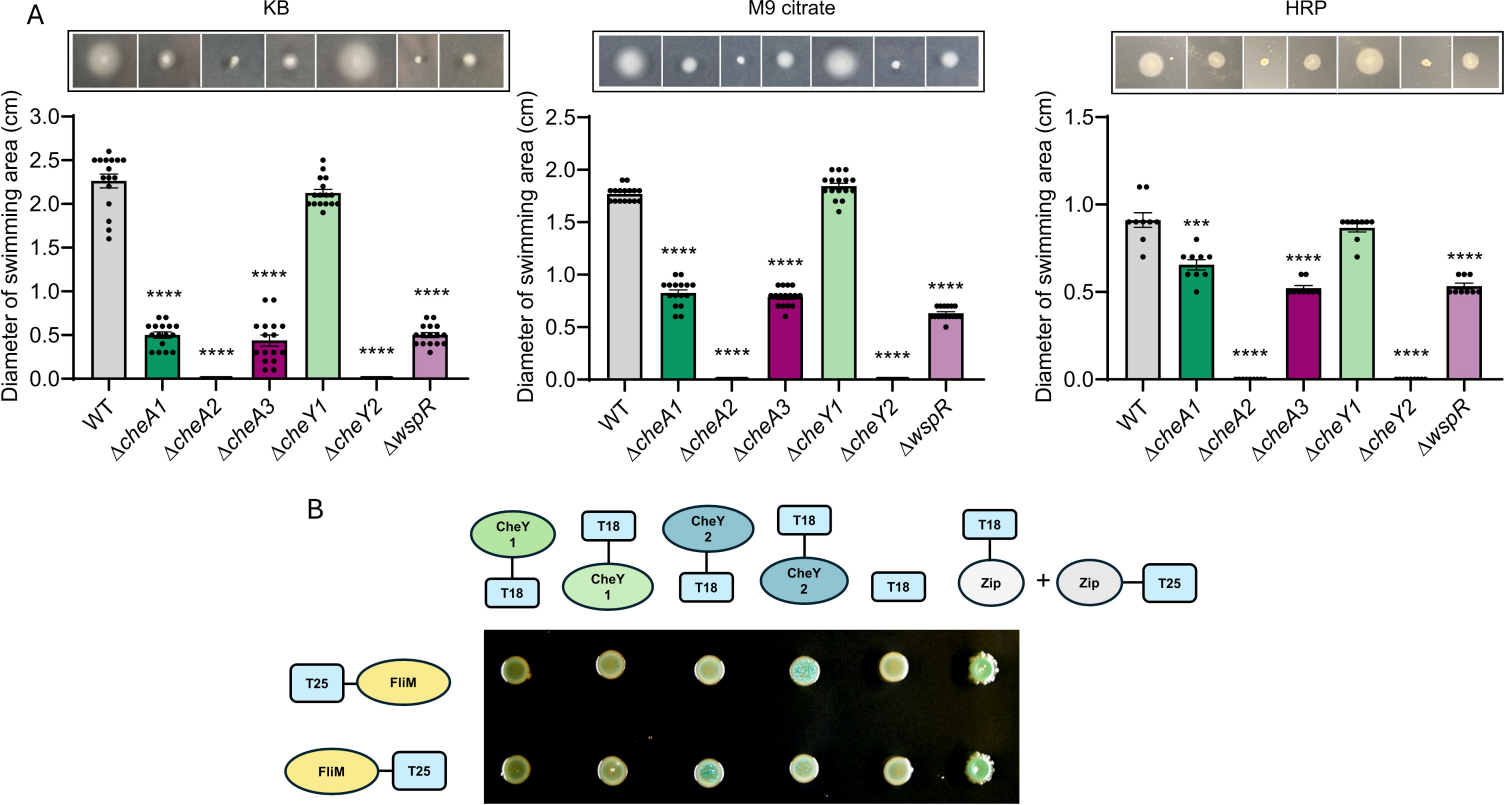
Swimming motility assays and protein interactions between FliM and CheY response regulators. (**A**) Swimming motility assays in KB, M9 citrate, or HRP medium agar plates (0.3%, wt/vol). Bars represent the means of four independent experiments with four technical replicates each. Values that are significantly different are indicated by asterisks (***P < 0.001 and ****P < 0.0001). (**B**) Bacterial adenylate cyclase two-hybrid assays between FliM and the CheY1 and CheY2 proteins from PsPto. Experiments were conducted in triplicate with three technical replicates each. For better visualization, only one replicate is shown for each interaction. A schematic representation of proteins cloned at the N-terminal or C-terminal of the T25 or T18 adenylate cyclase fragment is presented. FliM was tested against the T18 fragment of the adenylate cyclase as a negative control. Interaction between the leucine zipper of GCN4 in frame with the T25 and T18 fragments of the adenylate cyclase was used as a positive control.

To assess the role of the PsPto chemosensory systems in chemotaxis-related motility, we performed soft agar swimming assays in strains lacking either *CheA* or the corresponding response regulator in each chemosensory system (Fig. 4A). All strains lacking *CheA* showed swimming defects, but the deletion of *cheA2* completely abolished motility in all media tested. Δ*cheY2* displayed the same swimming phenotype as Δ*cheA2* in all media tested, and Δ*wspR* displayed a similar phenotype as Δ*cheA1* and Δ*cheA3,* showing a decreased swimming motility. However, Δ*cheY1* showed a similar motility phenotype to the WT strain in all media tested. Swimming was partially restored in the Δ*cheA2*-complemented strain and totally restored in the Δ*cheY2*-complemented strain (Fig. S2). Swimming complementation of Δ*cheA1*, Δ*cheA3,* and Δ*wspR* strains was not observed (data not shown).

CheY-type response regulators bind to the flagellar motor protein FliM to modulate flagellar rotation (68). Based on the two opposite motility phenotypes observed in the two PsPto Δ*cheY* mutants, we carried out bacterial adenylate-cyclase two-hybrid (BACTH) assays to test the potential interactions between PsPto FliM and both CheY proteins. Results showed that CheY2 interacts with FliM, while no binding was detected between CheY1 and FliM (Fig. 4B).

### Chemosensory systems contribute to biofilm-related phenotypes

**Fig 5:**
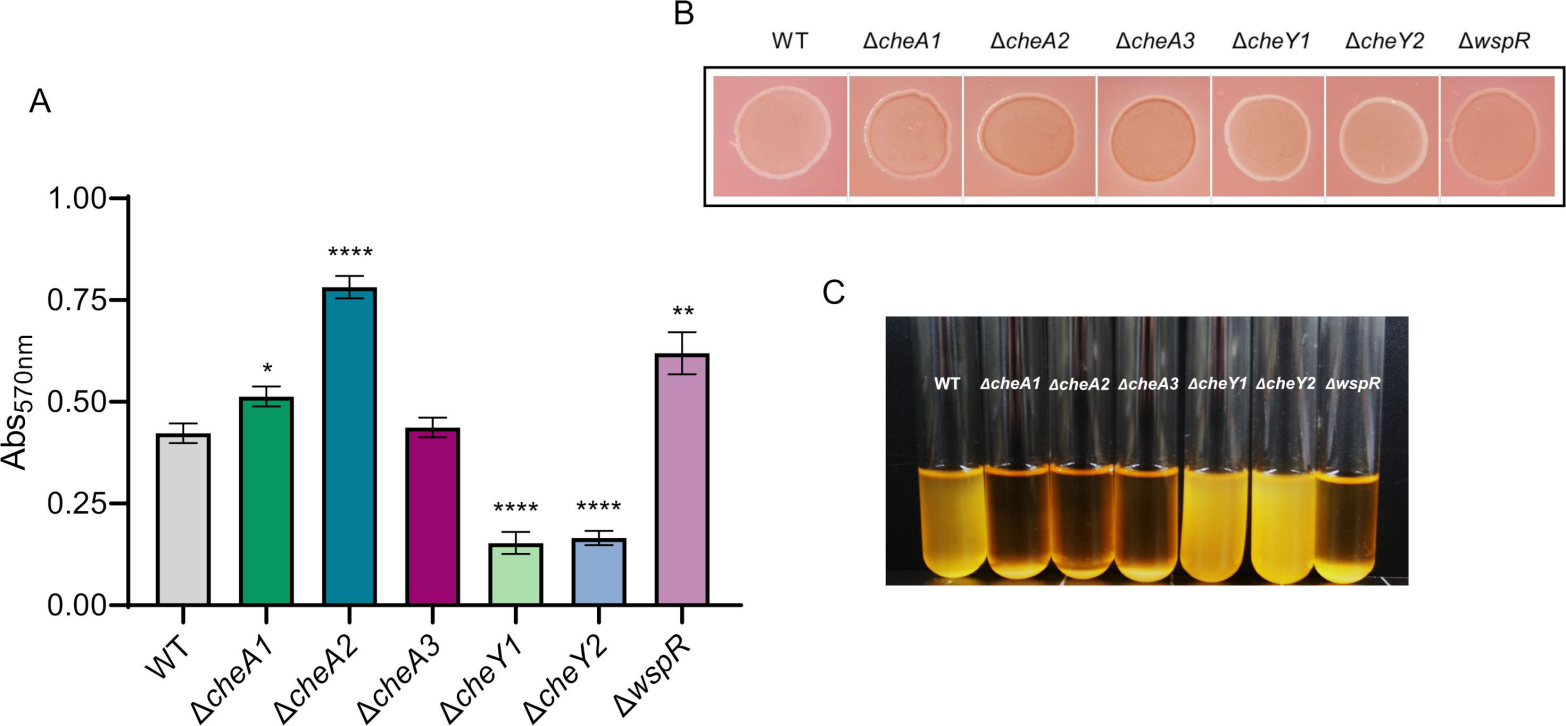
Biofilm-related traits. (**A**) Crystal violet attachment assay. Bars represent the means of three independent experiments with six technical replicates each. Values that are significantly different are indicated by asterisks (*P  <  0.05; **P  <  0.01; and ****P < 0.0001). (**B**) Representative image of the Congo red uptake by colonies grown on M9 citrate plates. Experiments were conducted in triplicate with six technical replicates each. (**C**) Flocculation assays of the WT and mutant strains. Overnight cultures were left to flocculate at room temperature overnight. Three independent experiments were conducted with three technical replicates each. For better visualization, only one replicate is shown for each strain. White arrow points to the sedimentation area.

Attachment assays were performed using the crystal violet method described previously (44) (Fig. 5A). Δ*cheA1,* Δ*cheA2*, and Δ*wspR* showed a significant increase in attachment when compared to the WT. Contrarily, Δ*cheY1* and Δ*cheY2* attached significantly less than the WT strain. A *cheA3* deletion did not cause any attachment alteration in this assay.

We decided to analyze the Congo red uptake ability, which is related to exopolysaccharide (EPS) production and indirectly to biofilm formation (69) (Fig. 5B). First, we observed that Δ*cheA1*, Δ*cheA2*, Δ*cheA3*, and Δ*wspR* mutants presented a higher Congo red uptake ability than the WT. In contrast, Δ*cheY1* and Δ*cheY2* showed Congo red uptake similar to that of the WT strain. Flocculation phenotype in liquid media is also related to EPS production, and we also observed that liquid cultures of Δ*cheA1*, Δ*cheA2*, Δ*cheA3*, and Δ*wspR* flocculated overnight when left static at room temperature, whereas the WT flocculated partially, and Δ*cheY1* and Δ*cheY2* remained homogeneously distributed across the liquid media (Fig. 5C). We also analyzed the colony morphology and texture in rich medium, which have also been related to the biofilm formation ability (70) (Fig. S3). We could observe how the inner colony areas of Δ*cheA1*, Δ*cheA2*, Δ*cheA3*, and Δ*wspR* exhibited more pronounced wrinkling as compared to the WT. In contrast, Δ*cheY1* and Δ*cheY2* displayed a smooth colony morphology.

### Bacterial two-hybrid assays reveal unusual interaction partners

**Fig 6:**
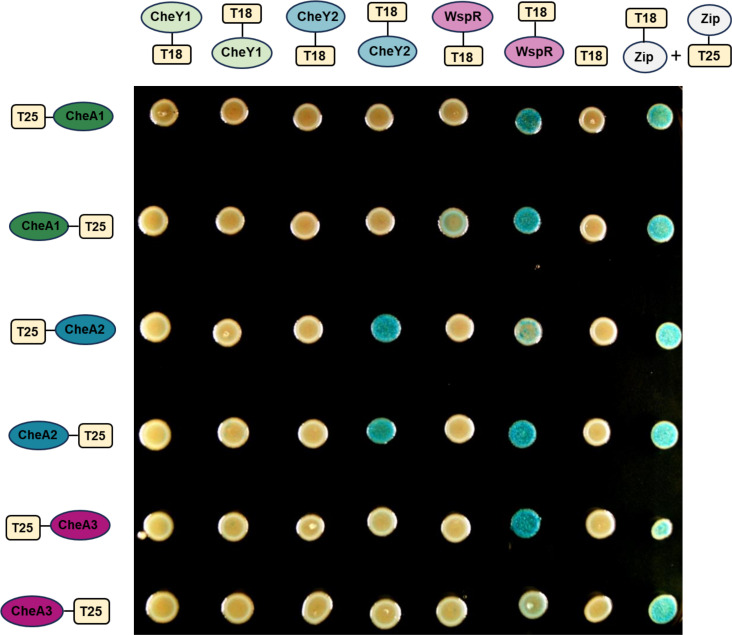
Bacterial adenylate cyclase two-hybrid assays between CheAs and response regulators from PsPto. Experiments were conducted in triplicate with three technical replicates each. For better visualization, only one replicate is shown for each interaction. A schematic representation of proteins cloned at N-terminal or C-terminal of the T25 or T18 adenylate cyclase fragment is presented. CheA1, CheA2, and CheA3 were tested against the T18 fragment of the adenylate cyclase as a negative control. Interaction between the leucine zipper of GCN4 in frame with the T25 and T18 fragments of the adenylate cyclase was used as a positive control.

The above-described results point to a possible crosstalk between the three chemosensory systems analyzed in this work (F6, F8, and ACF). Canonically, we would expect the kinase CheA2 to interact with the response regulator CheY2 (F6 system), CheA1 to interact with CheY1 (F8 system), and CheA3 to interact with WspR (ACF system). BACTH assays were performed to check interactions among the three kinases and the three response regulators. Results showed that, as expected, CheA2 and CheA3 interact with their canonical response regulators (Fig. 6). Surprisingly, we were not able to detect the interaction between CheA1 and CheY1 response regulator. Moreover, a clear interaction was observed in the case of both CheA1 and CheA2 with the response regulator WspR (Fig. 6).

### Chemosensory systems are necessary for an optimal plant entry to incite infection

**Fig 7:**
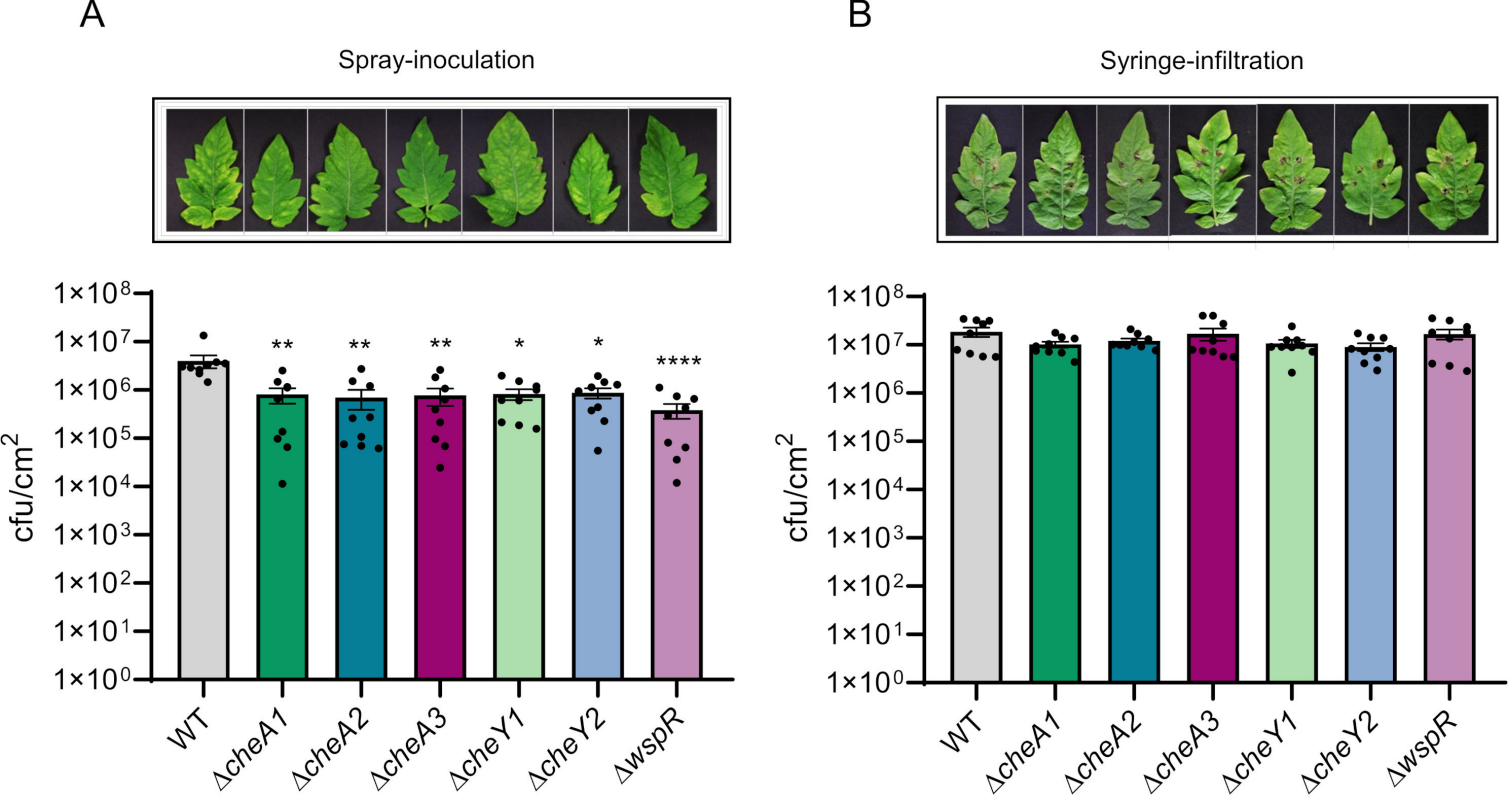
Plant virulence assays with two inoculation methods. (**A**) Spray-inoculated virulence assays of tomato plants. Bars represent bacterial populations of strains at 6 days post-inoculation (dpi) after spray inoculation (1 × 10^8^ CFU/mL) (**B**) Syringe infiltration virulence assay. Bars represent bacterial populations of strains at 3 dpi after syringe infiltration (3 × 10^4^ CFU/mL). Shown are means and standard errors from three independent biological replicates. In each experiment, three plants were analyzed by sampling five 1-cm-diameter leaf discs per plant. Values that are significantly different are indicated by asterisks (*P  <  0.05; **P  <  0.01; and ****P < 0.0001).

We tested the ability of PsPto chemosensory mutant strains to infect tomato plants. We used different inoculation methods, the spray-inoculation approach, which mimics natural entry conditions that bacteria face in the phyllosphere, and the infiltration method, in which the pathogen is placed inside the plant. Bacterial populations of mutant strains were reduced compared to that of the WT strain when cells were spray-inoculated. However, this difference was abolished when the strains were inoculated by infiltration (Fig. 7). Nevertheless, we could observe how syringe-inoculated tomato leaves with Δ*cheA3* and Δ*wspR* mutant strains displayed a decreased necrotic area than the WT strain (Fig. S4). These findings demonstrate how all three chemosensory systems play a key role during the early stages of the infection of tomato plants. Furthermore, the ACF system also seems to be involved in symptom development.

## DISCUSSION

Many bacteria have evolved more than one chemosensory system (10), which is arranged independently, and it has been proposed that this cluster-based scaffolding allows the spatial separation of chemotaxis pathways putatively involved in specific cellular functions (19).

PsPto has four main chemosensory clusters, namely, F6, F8, ACF, and Tfp systems, but many questions remain to be answered regarding the specific functions they control. Here, we have assigned all 49 chemoreceptors of PsPto to one of the four main chemosensory clusters. The assignment is based solely on a deductive process based on previous data and assignments in other organisms (64, 71). Therefore, further experimental data will be necessary to confirm or refute our assignment.

Most of the 40H chemoreceptors have been assigned to the F6 system. However, the 40H chemoreceptors PSPTO_1493 and PSPTO_5031 are proposed to be assigned to the ACF and Tfp systems, respectively. This assignment is based on both genomic context and experimental data on homologous chemoreceptors of PAO1 (65, 66). Regarding PSPTO_0912, the only 34H class chemoreceptor of PsPto, we proposed its assignment to the F8 system based on its genomic location. Moreover, it is the only chemoreceptor of PsPto containing a C-terminal pentapeptide, a domain predicted to interact with CheR proteins containing a GXX insertion (72). The only CheR in PsPto that presents this feature is also encoded in the F8 cluster (PSPTO_0910), which supports the assignment of PSPTO_0912 to this system. PSPTO_2441 is the only chemoreceptor of the 36H class. As chemoreceptors of 36H and 40H tend not to cluster together (17) and experimental data support (65) the assignment of PSPTO_5031 (40H) to the Tfp system, we could speculate that PSPTO_2441 is working with the F6 system instead (64).

Using cryo-electron tomography, we have been able to visualize two types of chemoreceptor arrays in PsPto, which correspond to arrays of the F6 and F8 classes. Surprisingly, in the Δ*cheA2* mutant background, we still observe the presence of F6 arrays. A similar result has been observed in the case of *Azospirillum brasilense* mutant strains lacking the Che1 and Che4 clusters, corresponding to F5 and F7 systems (23). In this case, the authors suggest the formation of mixed baseplates in the arrays, which would support the formation of the arrays in the absence of any of the CheA proteins (23). However, we cannot discard the formation of non-functional arrays in the absence of CheA2 due to the presence of the coupling protein CheW. Actually, array formation in the absence of CheA has been experimentally shown to be possible in the case of *Vibrio cholerae* (73).

Our results confirm that in PsPto, the F6 system is required for chemotaxis and in plate swimming motility. In contrast, the F8 and ACF systems of PsPto do not seem to be involved in the regulation of chemotaxis in capillary assays, but they have a function in motility modulation in plate swimming assays. These results are consistent with previous reports in other bacteria with more than one chemosensory system, showing the function of different chemotaxis pathways regulating distinct motility parameters. In *Rhizobium leguminosarum*, the Che1 pathway has been found to be the major chemotaxis cluster controlling motility pattern, while Che2 plays a minor role (74). In the case of *A. brasilense*, Che1 controls swimming speed and has only a minor role in chemotaxis, while the Che4 system controls cell tumbling, which is essential for chemotaxis (75). Our results on the main role of CheA2 kinase in swimming motility and, therefore, of the F6 chemosensory system are in line with previous results from Clarke et al. (45). However, we also found a role for the CheA1 and CheA3 kinases in the modulation of swimming motility that was not reported in this previous work. It is worth highlighting that the authors constructed two types of mutations, disruption and deletion mutants, and the described data are based on the disruption mutant phenotypes. We have constructed clean deletion mutants and observed no defects in growth in M9 citrate medium (Fig. S5). Moreover, in *P. syringae* pv. tabaci, a pathovar closer to PsPto, the key role of the CheA2 kinase in the control of both chemotaxis and swimming motility and a minor implication of the CheA1 kinase in these phenotypes (76) have been shown. However, there are no data on the putative function of the kinase from the ACF system in this pathovar. Notably, no complementation was possible in any of the previous works showing phenotypes for CheA1 homologs. This could be due to the nature of a tight regulation of kinase functions.

**Fig 8:**
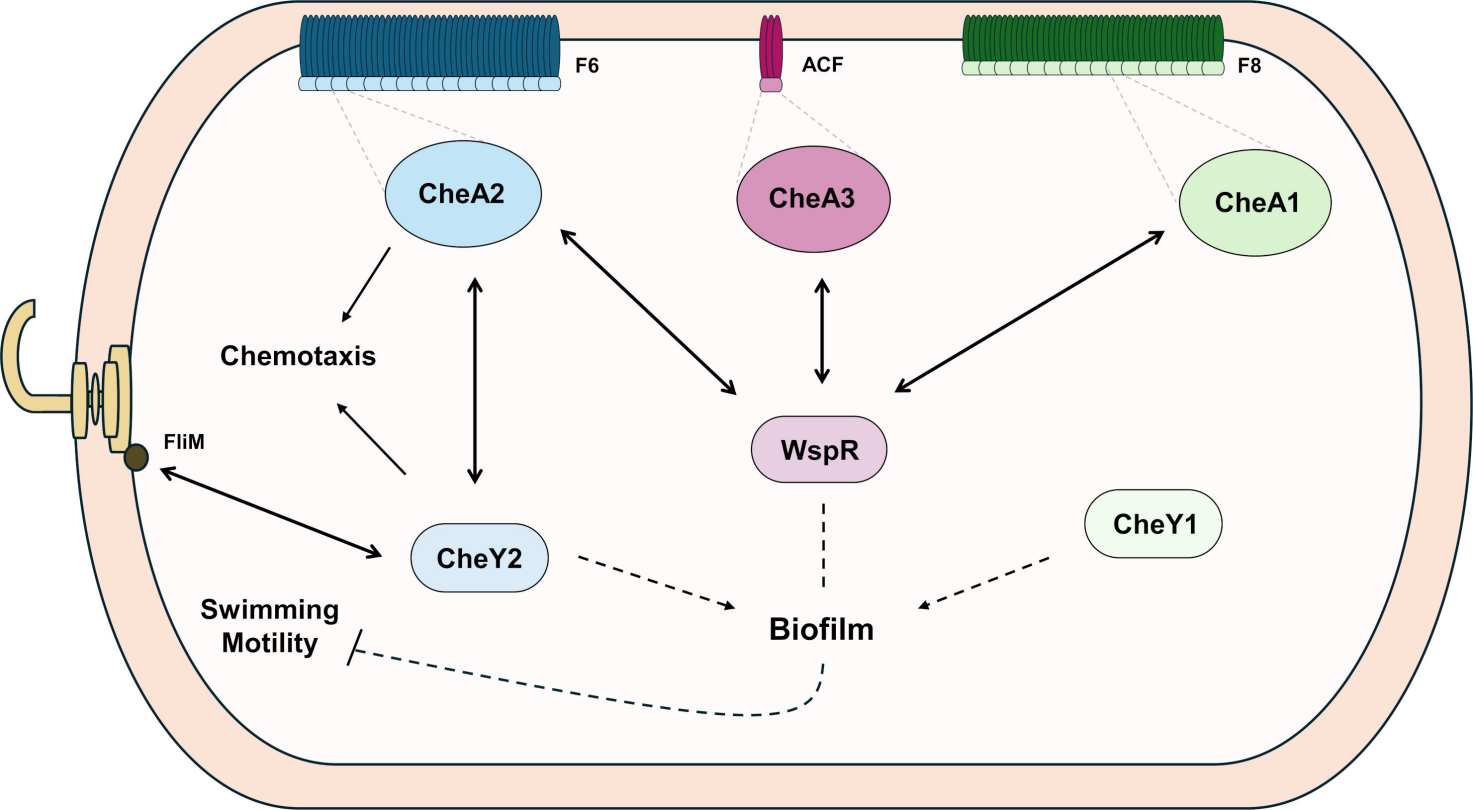
Proposed model of signal transduction and crosstalk among chemosensory systems in PsPto. Double-headed arrows indicate protein interaction. Solid lines indicate regulatory functions; arrow ends indicate promotion, and dash ends indicate suppression. Dashed arrows indicate regulatory functions through unknown elements; arrow ends indicate promotion, and dash ends indicate suppression.

Our observations reveal how CheY2 is the true chemotaxis response regulator and works together with CheA2 to ensure chemotaxis and swimming (Fig. 8). However, while CheY1 does not seem to have a role in motility, the CheA1 kinase contributes to the control of this trait (Fig. 8). This reveals how CheA1 and CheY1 work independently regarding motility. In bacteria containing more than one CheY homolog (9, 77, 78), it is common that one CheY homolog is essential for the chemotaxis response while the remaining CheY homolog works as a phosphate sink. This is the case for *Sinorhizobium meliloti* and *Azorhizobium caulinodans*. It is consistent that a mutation in the essential response regulator abolishes swimming behavior, while a mutation in the phosphate sink response regulator decreases swimming performance (77, 79). However, this does not seem to be the case in PsPto, as a Δ*cheY1* mutant does not show any defect in swimming motility. The dual role of two CheY homologs was also assessed in *Agrobacterium fabrum* (78). In this case, CheY1 and CheY2 from *A. fabrum* were both discovered to contribute to chemotaxis by binding FliM with different affinities, which culminated in different rates of swimming reversals, but both were shown to interact with the same CheA (78). In PsPto, we have shown the interaction between CheY2 and FliM, although the interaction between CheY1 and FliM could not be detected. This could explain the absence of a swimming phenotype in the Δ*cheY1* mutant.

Biofilm formation has been primarily described as a feature under the control of ACF systems. However, several reports over the last years have shown how F systems also contribute to the regulation of biofilm formation (20–23, 80). Indeed, we have observed an increased biofilm formation in Δ*cheA1* and Δ*cheA2* mutant strains, which belong to the F8 and F6 systems, respectively. The highest attachment capacity (which is the first step of biofilm formation) observed in the Δ*cheA2* mutant aligns with the phenotype observed in a *cheA* mutant strain of *E. coli* (81). Moreover, these observations are in line with the findings in PAO1 where the CheA kinase has been described to interact with the biofilm dispersal protein DipA phosphodiesterase involved in the control of c-di-GMP homeostasis (82).

Surprisingly, in terms of biofilm-related traits, mutants in the CheY response regulators displayed opposed phenotypes than those of the mutants in kinases encoded in the same clusters. This suggests that CheYs work independently from their cognate kinases to promote biofilm formation (Fig. 8). A similar situation was reported in *Shewanella oneidensis*, where it was shown that phosphorylated CheY3 is essential for biofilm formation and that its phosphorylation comes from a different kinase than its cognate kinase CheA3 (83). They also showed that CheY3 interacts with Mxd proteins and the diguanylate cyclase PdgB. Mxd proteins are c-di-GMP effectors that belong to the glycosyl transferase family and are involved in polysaccharide production (84). Other signaling crosstalk has been proposed for *Agrobacterium tumefaciens*, where its CheY2 response regulator would be additionally phosphorylated by a different kinase than its own cognate kinase to modulate flagellar motility (85). Phenotypes of Δ*cheY* mutants in PsPto correlate with low levels of intracellular c-di-GMP, suggesting their role in the control of the level of this second messenger. Low levels of c-di-GMP levels correlate with decreased biofilm and high motility, as the expression of flagellar genes and biofilm-related genes is inversely regulated (86, 87). Another explanation for the decreased biofilm observed in the Δ*cheY2* mutant is that, since the transfer of the phosphoric group to the primary target is abolished, the CheA2 kinase could activate other response regulators like WspR. This is supported by the interaction observed between CheA2 and WspR. It is important to note that this finding does not contradict the impaired motility of the Δ*cheY2* mutant. It would be expected that a strain with low levels of c-di-GMP had an increased motility. However, the lack of CheY2 impedes the transduction of signaling to the flagellar motor at the level of the interaction with FliM, resulting in a non-chemotactic strain regardless of its c-di-GMP levels.

Phenotypes of the Δ*wspR* mutant strain correlate with those of the Δ*cheA3* mutant strain, which relate with high c-di-GMP levels, suggesting a tandem activity of CheA3 kinase-WspR response regulator. Extensive work has been done in *Pseudomonas*, showing the WspR diguanylate cyclase activity as a response to the stimulation of the WspA chemoreceptor, resulting in increased c-di-GMP levels and biofilm formation (4, 66, 88–91). Our work about the ACF system does not align with most of the literature available on the topic. Armbruster et al. (92) reported that a deletion of *wspR* in *P. aeruginosa* resulted in similar amounts of biofilm relative to WT at later stages (24 hours), suggesting that any of the many other c-di-GMP cyclases present in this bacterium may ultimately compensate for c-di-GMP production in the absence of WspR (92). In the PsPto genome, there are 56 genes encoding putative c-di-GMP cyclases (93). These activities could account for a compensation activity in the Δ*wspR* mutant background. Consequently, they might also contribute to compensatory mechanisms in the Δ*cheA3* mutant, where the signaling pathway is impaired. However, other regulatory mechanisms leading to the modulation of the levels of c-di-GMP could be taking place.

BACTH analysis revealed unexpected interactions of WspR with both CheA1 and CheA2, which could explain the enhanced biofilm-related phenotypes observed in the Δ*cheA1* and Δ*cheA2* mutants. As in the case of the signaling through CheA3, if these two kinases also participate in keeping the homeostasis of c-di-GMP through WspR, their mutation could induce a compensatory effect. However, we cannot discard interactions between CheAs and other biofilm regulators. Previous data on *Comamonas testosteroni* also reveal the interaction of the chemotaxis kinase with two different response regulators, the chemotaxis response regulator CheY and the biofilm response regulator FlmD (22). We were not able to detect the interaction between the kinase CheA1 and the response regulator CheY1 in our assays. This fact, together with the independency in the control of phenotypes observed in mutant strains in these two proteins, suggests that they do not collaborate in the same signaling pathway (Fig. 8).

Our results showed that both the kinases and the response regulators belonging to F6, F8, and ACF pathways are needed for full virulence in tomato plants. All chemosensory systems are relevant in the stage involving an active entry process, as syringe-infiltration assays restored *in planta* bacterial population of mutant strains. However, the ACF system seems to have an additional role involving symptom development, as leaves inoculated with chemosensory mutants displayed reduced necrotic areas when compared to the WT. All mutant strains except Δ*cheY1* are affected in their swimming abilities. This explains the defect in virulence, as PsPto requires active flagellar motility to achieve a successful infection/trigger infection (94). Δ*cheY1* did not show a defect in motility but a phenotype in biofilm formation. Since PsPto is a weak epiphyte, we could hypothesize that the epiphytic fitness of this strain is further compromised, rendering a lower infection ability.

We proposed a model of signal transduction and crosstalk among chemosensory systems in PsPto (Fig. 8). CheA2 would signal through the response regulator CheY2 to modulate flagellar rotation through its interaction with FliM. Furthermore, CheA1, CheA2, and CheA3 would also interact with WspR, regulating biofilm formation and swimming. CheA1, CheA3, and WspR functions could modulate motility through indirect intermediates, such as c-di-GMP levels (89, 90). Our results also indicate how CheY1 and CheY2 response regulators do not align with their cognate kinases to regulate biofilm formation, where they act as biofilm promoters. These data suggest that both kinases and response regulators of PsPto interact with additional partners to control key traits involved in virulence. Further analyses are needed to unveil the complex regulation network associated with chemosensory functions in this bacterium.

Overall, we show for the first time the visualization of the F6 and F8 chemosensory systems of PsPto. Most of the chemoreceptors are assigned to the F6 system, which supports the chance for this visualization. The visualization of the F8 chemosensory system suggests that the only CR protein known to associate with it must have quite a relevant role in PsPto. Moreover, our results unveil the crosstalk among the F6, F8, and ACF systems at the level of the interactions between the CheA1 and CheA2 kinases with the response regulator WspR, pointing to this response regulator as a central sink of the chemosensory functions in PsPto for the control of virulence and related traits. This work shows novel results that contribute to the knowledge of chemosensory systems and their role in functions alternative to chemotaxis.

## SUPPLEMENTAL MATERIAL

Supplemental material mbio.00871-24-s0001.docx Extended materials and methods; supplemental tables and figures.

ASM does not own the copyrights to Supplemental Material that may be linked to, or accessed through, an article. The authors have granted ASM a non-exclusive, world-wide license to publish the Supplemental Material files. Please contact the corresponding author directly for reuse.
