# Supplementary material for: Chemosensory systems interact to shape relevant traits for bacterial plant pathogenesis
Source: mBio. 2024 Jun 20;15(7):e00871-24. doi: 10.1128/mbio.00871-24 (PMC11253619; doi:10.1128/mbio.00871-24)
Supplement: Supplemental material — Extended materials and methods; supplemental tables and figures. [file mbio.00871-24-s0001.docx]

Supplementary information – Text S1

Content of Text S1

1. Supplemental material and methods
2. Construction of deletion mutant strains
3. Construction of complemented strains
4. Attachment assays
5. Bacterial Two-Hybrid Assays
6. Virulence assay
7. Growth curves
8. References
9. **Supplemental materials and methods**
10. **Construction of deletion mutant strains**

Mutagenesis in PsPto was performed by deletion of whole genes or internal regions of a given gene, using the small, mobilizable, sucrose-counter selection vector pK18mobsac (1). Approximately 1-kb fragments corresponding to the upstream and downstream region of the target gene were PCR amplified and fused together by an overlap extension PCR procedure. This resulting 2-kb fragment was cloned into XmaI sites of pK18mobsac and checked by PCR and sequencing.

pK18mobsac::Δ*target_gene* was introduced into PsPto by electroporation and first recombination events were selected with kanamycin. Colonies which were kanamycin resistant were picked and diluted into 1 ml of KB medium. 50 to 200 microliters were plated on KB plates supplemented with 10% sucrose to select for second recombination events. Colonies that survived sucrose selection were tested for kanamycin sensitivity, and resulting colonies were checked by PCR.

1. **Construction of complemented strains**

Plasmids for complementation assays were constructed using the pBBR1MCS2 (2). Target genes includingtheir promoter region, when possible, were PCR amplified and cloned into XmaI sites of pBBR1MCS2. Insert orientation was checked by PCR and subsequently sequenced.

The Δ*cheA2* was complemented using the pME6010::CheA2 plasmid, kindly given by Clarke et al., 2016.

1. **Attachment assays**

Overnight cultures grown in KB liquid medium at 28ºC were washed three times in MGA liquid medium (54 mM mannitol, 3.6 mM KH_2_PO_4_, 23 mM NaCl, 0.8 mM MgSO_4_, 18 mM NH_4_Cl; pH 7.0) supplemented with 2 mM CaCl_2_ and adjusted to a final OD_600_ of 0.02. 100 µl of bacterial suspensions were added to 96-well plates and incubated statically at room temperature for 48 h. After 48 h, planktonic cells were removed from wells and wells were gently washed 3 times with distilled water. 150 µl of 0.1% (wt/vol) crystal violet (CV) stain was added to each well and incubated for 30 min. After three gentle washes with distilled water, stained biofilms were resuspended in 30% (vol/vol) acetic acid. The absorbance at OD_570_ was recorded for each well and the average and standard deviation for technical replicates were computed.

1. **Bacterial two-hybrid assays**

Bacterial strains and plasmids were purchased from Euromedex (France). The reporter strain *E. coli* DHM10 (cya^-^) was co-transformed with two-hybrid plasmids. For negative controls, pKT25-CheA1, pKT25-CheA2, pKT25-CheA3, pKT25-FliM, and pUT18 were used. For positive controls, pKT25-zip and pUT18-zip were used. Clones were selected on LB agar plates supplemented with ampicillin (100 µg/ml) and kanamycin (50 µg/ml). Three clones of each co-transformation were inoculated in LB medium supplemented with kanamycin (50 µg/ml) and ampicillin (100 µg/ml) and grown at 37ºC and 200 r.p.m. for at least 4 hours. 2 µl of bacterial culture were spotted either on LB plates supplemented with kanamycin (50 µg/ml), ampicillin (100 µg/ml), 0.5 mM IPTG, and X-gal (40 µg/ml). Plates were incubated at RT or 28ºC until colonies turned blue.

1. **Virulence assays**

PsPto strains were grown at 28°C for 24 h on KB agar in darkness. Cells were resuspended in 10 mM MgCl_2_ and diluted to 10^8^ CFU/ml. For spray inoculation assays, three-week-old tomato plants (*Solanum lycopersicum* cv. Moneymaker) were sprayed with a suspension containing 10^8^ CFU/ml, and silwet L-77 was added to the bacterial suspensions at a final concentration of 0.02% (vol/vol). Plants were incubated in a growth chamber at 25°C and 60% RH with a daily light period of 12 hours. Six days after inoculation, the leaf symptoms were recorded, and bacterial populations from three plants were measured by sampling five 1-cm-diameter leaf disks per plant. The infected leaf disks were washed twice with 10 mM MgCl_2_ prior to homogenization to eliminate the bacteria from the leaf surface. Plant material was homogenized in 10 mM MgCl_2_ and drop plated onto KB agar supplemented with the appropriate antibiotics. The average number of bacteria per square centimeter isolated from five infected tomato leaves was determined based on log-transformed data.

For syringe-infiltration assays, cells were resuspended in 10 mM MgCl_2_ and diluted to 3x10^4^ CFU/ml. Tomato leaves were pierced 4 times with a needle on the adaxial side of leaves and 100 µl of bacterial suspension were syringe-infiltrated through the wound. Plants were incubated in a growth chamber at 25ºC and 60% RH with a daily period of 12 hours. Three days after inoculation, the leaf symptoms were recorded, and bacterial populations were determined in the same way as in spray inoculation assays.

1. **Growth curves**

Bacterial strains were grown overnight in KB medium, washed twice with M9 10 mM citrate and adjusted to an OD_600_ of 0.05 in a final volume of 50 ml. Measurements were performed every hour for 7 timepoints, and a final measurement was taken at 24 hours.

1. **References**

1. Kvitko BH, Collmer A. 2011. Construction of *Pseudomonas syringae* pv. tomato DC3000 mutant and polymutant strains. Methods Mol Biol 712:109-128.

2. Kovach ME, Elzer PH, Hill DS, Robertson GT, Farris MA, Roop RM, Peterson KM. 1995. Four new derivatives of the broad-host-range cloning vector pBBR1MCS carrying different antibiotic-resistance cassettes. Gene 166:175-176.

Supplementary information – Tables

| **Table S1.** Bacteria and plasmids used in this study. | | |
| --- | --- | --- |
| **Strains and plasmids** | **Relevant characteristics ^a^** | **Reference or source** |
| ***E. coli*** |  |  |
| DH5α | *supE*44 *lacU*169 (*Ø80lacZ*ΔM15) *hsdR*17 (r_k_-m_k_-) *recA*1 *endA*1 *gyrA*96 *thi*-1 *relA*1 | (1) |
| DHM1 | *F- cya-854 recA1 endA1 gyrA96 (Nal r) thi1 hsdR17 spoT1 rfbD1 glnV44(AS)* | Euromedex, France |
| ***P. syringae* pv. tomato DC3000** |  |  |
| PsPto | Rif^r^, wild type | (2) |
| Δ*cheA1* | Deletion of the first 1,032 bp from the [*PSPTO_0913*](https://www.ncbi.nlm.nih.gov/gene/?term=PSPTO_0913) gene from PsPto | This study |
| Δ*cheA2* | Deletion of the first 1,214 bp from the [*PSPTO_1982*](https://www.ncbi.nlm.nih.gov/gene/?term=PSPTO_1982) gene from PsPto | This study |
| Δ*cheA3* | In-frame deletion of 2,343 bp from the [*PSPTO_1497*](https://www.ncbi.nlm.nih.gov/gene/?term=PSPTO_1497) gene from PsPto | This study |
| Δ*cheY1* | In-frame deletion of 348 bp from the [*PSPTO_0910*](https://www.ncbi.nlm.nih.gov/gene/?term=PSPTO_0910) gene from PsPto | This study |
| Δ*cheY2* | In-frame deletion of 336 bp from the [*PSPTO_1980*](https://www.ncbi.nlm.nih.gov/gene/?term=PSPTO_1980) gene from PsPto | This study |
| Δ*wspR* | Deletion of the complete [*PSPTO_1980*](https://www.ncbi.nlm.nih.gov/gene/?term=PSPTO_1980) gene and 18 bp of its intergenic region from PsPto | This study |
| PsPto-pBBR1MCS-2 | PsPto harboring the pBBR1MCS-2 plasmid | This study |
| Δ*cheA2*-C | Δ*cheA2* harboring wild type *cheA2* in plasmid; pME6010::CheA2; Tc^r^ | This study |
| Δ*cheY2*-C | Δ*cheY2* harboring wild type *cheY2* in plasmid; pBBR1MCS2::CheY2; Km^r^ | This study |
| **Plasmids** |  |  |
| pK18mobsac | Km^r^ oriColE1 *sacB mob*RP4 | (3) |
| pK18mobsac::*cheA1-KO* | Km^r^, pK18mobsac plasmid harboring 1-kb upstream and 1-kb downstream of an internal region of *cheA1* gene | This study |
| pK18mobsac::*cheA2-KO* | Km^r^, pK18mobsac plasmid harboring 1-kb upstream and 1-kb downstream of the *cheA2* gene | This study |
| pK18mobsac::*cheA3-KO* | Km^r^, pK18mobsac plasmid harboring 1-kb upstream and 1-kb downstream of the *cheA3* gene | This study |
| pK18mobsac::*cheY1-KO* | Km^r^, pK18mobsac plasmid harboring 1-kb upstream and 1-kb downstream of the *cheY1* gene | This study |
| pK18mobsac::*cheY2-KO* | Km^r^, pK18mobsac plasmid harboring 1-kb upstream and 1-kb downstream of the *cheY2* gene | This study |
| pK18mobsac::*wspR-KO* | Km^r^, pK18mobsac plasmid harboring 1-kb upstream and 1-kb downstream of the *cheY2* gene | This study |
| pBBR1MCS-2 | Km^r^, *ori*RK2 *mob*RK2 | (4) |
| pBBR1MCS-2::CheY2 | Km^r^; a 615-bp PCR fragment containing the [*PSPTO_1980*](https://www.ncbi.nlm.nih.gov/gene/?term=PSPTO_1980) gene and its promoter region | This study |
| pME6010::CheA2 | Tc^r^; a 2.3-kb PCR fragment containing the [*PSPTO_1982*](https://www.ncbi.nlm.nih.gov/gene/?term=PSPTO_1982) gene and its promoter region | (5) |
|  |  |  |
| pKT25 | Km^R^ plasmid encoding the T25 fragment of *Bordetella pertussis* *cyaA* under P_lac_ control and a downstream multiple cloning site (MCS) | Euromedex, France |
| pKT25-Zip | Leucine zipper of GCN4 (8) genetically fused in frame to the T25 fragment in the *Kpn*I site of pKT25. | Euromedex, France |
| pKT25-CheA1 | *cheA1* ([*PSPTO_0913*](https://www.ncbi.nlm.nih.gov/gene/?term=PSPTO_0913)) was amplified from PsPto DNA and cloned into the *Pst*I site of pKT25 and checked for correct orientation to express T25-CheA1 | This work |
| pKT25-CheA2 | *cheA2* ([*PSPTO_1982*](https://www.ncbi.nlm.nih.gov/gene/?term=PSPTO_1982)) was synthesized de novo (biocat) and cloned into the *Xba*I and *Bam*HI sites of pKT25 to express T25-CheA2 | This work |
| pKT25-CheA3 | *cheA3* ([*PSPTO_1497*](https://www.ncbi.nlm.nih.gov/gene/?term=PSPTO_1497)) was amplified from PsPto DNA and cloned into the *Bam*HI sites of pKT25 and checked for correct orientation to express T25-CheA3 | This work |
| pKT25-FliM | *fliM* ([*PSPTO_1969*](https://www.ncbi.nlm.nih.gov/gene/?term=PSPTO_1969)) was amplified from PsPto DNA and cloned into the *Bam*HI sites of pKT25 and checked for correct orientation to express T25-FliM | This work |
| pKNT25 | Km^R^ plasmid with Plac containing a MCS upstream of the T25 fragment of *Bordetella pertussis* *cyaA* | Euromedex, France |
| pKNT25-CheA1 | *cheA1* ([*PSPTO_0913*](https://www.ncbi.nlm.nih.gov/gene/?term=PSPTO_0913)) was synthesized de novo (Biocat) and cloned into the *Xba*I and *Bam*HI sites of pKT25 to express CheA1-T25 | This work |
| pKNT25-CheA2 | *cheA2* ([*PSPTO_1982*](https://www.ncbi.nlm.nih.gov/gene/?term=PSPTO_1982)) was synthesized de novo (Biocat) and cloned into the *Xba*I and *Bam*HI sites of pKT25 to express CheA2-T25 | This work |
| pKNT25-CheA3 | *cheA3* ([*PSPTO_1497*](https://www.ncbi.nlm.nih.gov/gene/?term=PSPTO_1497)) was amplified from PsPto DNA and cloned into the *Bam*HI sites of pKNT25 and checked for correct orientation to express CheA3-T25 | This work |
| pKNT25-FliM | *fliM* ([*PSPTO_1969*](https://www.ncbi.nlm.nih.gov/gene/?term=PSPTO_1969)) was amplified from PsPto DNA and cloned into the *Bam*HI sites of pKNT25 and checked for correct orientation to express FliM-T25 | This work |
| pUT18 | Ap^R^ plasmid with P_lac_ containing a MCS upstream of the T18 fragment of *Bordetella pertussis* *cyaA* | Euromedex, France |
| pUT18-CheY1 | *cheY1* ([*PSPTO_0910*](https://www.ncbi.nlm.nih.gov/gene/?term=PSPTO_0910)) was amplified from PsPto DNA and cloned into the *Bam*HI sites of pUT18 and checked for correct orientation to express CheY1-T18 | This work |
| pUT18-CheY2 | *cheY2* ([*PSPTO_1980*](https://www.ncbi.nlm.nih.gov/gene/?term=PSPTO_1980)) was amplified from PsPto DNA and cloned into the *Bam*HI sites of pUT18 and checked for correct orientation to express CheY2-T18 | This work |
| pUT18-WspR | *wspR* ([*PSPTO_1499*](https://www.ncbi.nlm.nih.gov/gene/?term=PSPTO_1499)) was amplified from PsPto DNA and cloned into the *Bam*HI sites of pUT18 and checked for correct orientation to express WspR-T18 | This work |
| pUT18C | Ap^R^ plasmid with Plac encoding the T18 fragment of *B. pertussis cyaA* and a downstream MCS | Euromedex, France |
| pUT18C-zip | Leucine zipper of GCN4 (6) genetically fused in frame to the T18 fragment in the *Kpn*I and *Eco*R1 sites of pUT18. | Euromedex. France |
| pUT18C-CheY1 | *cheY1* ([*PSPTO_0910*](https://www.ncbi.nlm.nih.gov/gene/?term=PSPTO_0910)) was amplified from PsPto DNA and cloned into the *Bam*HI sites of pUT18C and checked for correct orientation to express T18-CheY1 | This work |
| pUT18C-CheY2 | *cheY2* ([*PSPTO_1980*](https://www.ncbi.nlm.nih.gov/gene/?term=PSPTO_1980)) was amplified from PsPto DNA and cloned into the *Bam*HI sites of pUTC18 and checked for correct orientation to express T18-CheY2 | This work |
| pUT18C-WspR | *wspR* ([*PSPTO_1499*](https://www.ncbi.nlm.nih.gov/gene/?term=PSPTO_1499)) was amplified from PsPto DNA and cloned into the *Bam*HI sites of pUTC18 and checked for correct orientation to express T18-WspR | This work |
| 1. Ap; ampicillin, Km: kanamycin, Rf: rifampicin, Tc: tetracycline | | |

**References**

1. Hanahan D. 1983. Studies on transformation of *Escherichia coli* with plasmids. J Mol Biol 166:557-580.

2. Cuppels DA. 1986. Generation and characterization of Tn5 insertion mutations in *Pseudomonas syringae* pv. tomato. Appl Environ Microbiol 51:323-327.

3. Kvitko, B H, Collmer A. 2011. Construction of *Pseudomonas syringae* pv. tomato DC3000 mutant and polymutant strains. Methods Mol Biol 712:109-128..

4. Kovach ME, Elzer PH, Hill DS, Robertson GT, Farris MA, Roop RM, Peterson KM. 1995. Four new derivatives of the broad-host-range cloning vector pBBR1MCS carrying different antibiotic-resistance cassettes. Gene 166:175-176.

5. Clarke CR, Hayes BW, Runde BJ, Markel E, Swingle BM, Vinatzer BA. 2016. Comparative genomics of *Pseudomonas syringae* pathovar tomato reveals novel chemotaxis pathways associated with motility and plant pathogenicity. PeerJ 25:4:e2570.

6. Karimova G, Pidoux J, Ullmann A, Ladant D. 1998. A bacterial two-hybrid system based on a reconstituted signal transduction pathway. Proc Natl Acad Sci U S A 95(10):5752-6.

**Table S2.** Chemoreceptors of *Pseudomonas syringae* pv. tomato DC3000

| **Chemosensory system** | **MCP** | **LBD-domain** | **Class (Heptads)** | **C-terminal pentapeptide** | **Transmembrane domain** |
| --- | --- | --- | --- | --- | --- |
| F6 | [PSPTO_0466](https://www.ncbi.nlm.nih.gov/gene/?term=PSPTO_0466) | 4HB_MCP_1 | 40H | - | Yes |
|  | [PSPTO_0995](https://www.ncbi.nlm.nih.gov/gene/?term=PSPTO_0995) |  |  |  |  |
|  | [PSPTO_1334](https://www.ncbi.nlm.nih.gov/gene/?term=PSPTO_1334) |  |  |  |  |
|  | [PSPTO_2997](https://www.ncbi.nlm.nih.gov/gene/?term=PSPTO_2997) |  |  |  |  |
|  | [PSPTO_3279](https://www.ncbi.nlm.nih.gov/gene/?term=PSPTO_3279) |  |  |  |  |
|  | [PSPTO_3291](https://www.ncbi.nlm.nih.gov/gene/?term=PSPTO_3291) |  |  |  |  |
|  | [PSPTO_3577](https://www.ncbi.nlm.nih.gov/gene/?term=PSPTO_3577) |  |  |  |  |
|  | [PSPTO_3580](https://www.ncbi.nlm.nih.gov/gene/?term=PSPTO_3580) |  |  |  |  |
|  | [PSPTO_3685](https://www.ncbi.nlm.nih.gov/gene/?term=PSPTO_3685) |  |  |  |  |
|  | [PSPTO_4541](https://www.ncbi.nlm.nih.gov/gene/?term=PSPTO_4541) |  |  |  |  |
|  | [PSPTO_4624](https://www.ncbi.nlm.nih.gov/gene/?term=PSPTO_4624) |  |  |  |  |
|  | [PSPTO_5569](https://www.ncbi.nlm.nih.gov/gene/?term=PSPTO_5569) |  |  |  |  |
|  | [PSPTO_1061](https://www.ncbi.nlm.nih.gov/gene/?term=PSPTO_1061) | dCache_1 |  |  |  |
|  | [PSPTO_2448](https://www.ncbi.nlm.nih.gov/gene/?term=PSPTO_2448) |  |  |  |  |
|  | [PSPTO_2480](https://www.ncbi.nlm.nih.gov/gene/?term=PSPTO_2480) |  |  |  |  |
|  | [PSPTO_3237](https://www.ncbi.nlm.nih.gov/gene/?term=PSPTO_3237) |  |  |  |  |
|  | [PSPTO_1059](https://www.ncbi.nlm.nih.gov/gene/?term=PSPTO_1059) | HBM |  |  |  |
|  | [PSPTO_2616](https://www.ncbi.nlm.nih.gov/gene/?term=PSPTO_2616) |  |  |  |  |
|  | [PSPTO_3098](https://www.ncbi.nlm.nih.gov/gene/?term=PSPTO_3098) |  |  |  |  |
|  | [PSPTO_3680](https://www.ncbi.nlm.nih.gov/gene/?term=PSPTO_3680) |  |  |  |  |
|  | [PSPTO_5159](https://www.ncbi.nlm.nih.gov/gene/?term=PSPTO_5159) |  |  |  |  |
|  | [PSPTO_5160](https://www.ncbi.nlm.nih.gov/gene/?term=PSPTO_5160) |  |  |  |  |
|  | [PSPTO_5553](https://www.ncbi.nlm.nih.gov/gene/?term=PSPTO_5553) |  |  |  |  |
|  | [PSPTO_0263](https://www.ncbi.nlm.nih.gov/gene/?term=PSPTO_0263) | No LBD |  |  |  |
|  | [PSPTO_0916](https://www.ncbi.nlm.nih.gov/gene/?term=PSPTO_0916) |  | 24H |  |  |
|  | [PSPTO_2475](https://www.ncbi.nlm.nih.gov/gene/?term=PSPTO_2475) |  | 40H |  |  |
|  | [PSPTO_3480](https://www.ncbi.nlm.nih.gov/gene/?term=PSPTO_3480) |  |  |  |  |
|  | [PSPTO_3379](https://www.ncbi.nlm.nih.gov/gene/?term=PSPTO_3379) | sCache_2 |  |  |  |
|  | [PSPTO_3699](https://www.ncbi.nlm.nih.gov/gene/?term=PSPTO_3699) |  |  |  |  |
|  | [PSPTO_1066](https://www.ncbi.nlm.nih.gov/gene/?term=PSPTO_1066) | Unknown |  |  |  |
|  | [PSPTO_2254](https://www.ncbi.nlm.nih.gov/gene/?term=PSPTO_2254) |  |  |  |  |
|  | [PSPTO_2472](https://www.ncbi.nlm.nih.gov/gene/?term=PSPTO_2472) |  |  |  |  |
|  | [PSPTO_2526](https://www.ncbi.nlm.nih.gov/gene/?term=PSPTO_2526) |  |  |  |  |
|  | [PSPTO_4936](https://www.ncbi.nlm.nih.gov/gene/?term=PSPTO_4963) |  |  |  |  |
|  | [PSPTO_5554](https://www.ncbi.nlm.nih.gov/gene/?term=PSPTO_5554) |  |  |  |  |
|  | [PSPTO_1648](https://www.ncbi.nlm.nih.gov/gene/?term=PSPTO_1648) | PAS_3 |  |  |  |
|  | [PSPTO_2014](https://www.ncbi.nlm.nih.gov/gene/?term=PSPTO_2014) |  |  |  |  |
|  | [PSPTO_4531](https://www.ncbi.nlm.nih.gov/gene/?term=PSPTO_4531) | PAS_3-PAS_3 |  |  |  |
|  | [PSPTO_2511](https://www.ncbi.nlm.nih.gov/gene/?term=PSPTO_2511) | NIT |  |  |  |
|  | [PSPTO_0117](https://www.ncbi.nlm.nih.gov/gene/?term=PSPTO_0117) | Cache_3-Cache_2 |  |  |  |
|  | [PSPTO_1008](https://www.ncbi.nlm.nih.gov/gene/?term=PSPTO_1008) | PAS_3-PAS_3 | 24H |  | No |
|  | [PSPTO_5352](https://www.ncbi.nlm.nih.gov/gene/?term=PSPTO_5352) |  |  |  |  |
|  | [PSPTO_3753](https://www.ncbi.nlm.nih.gov/gene/?term=PSPTO_3753) | PAS_9-PAS_3 |  |  |  |
|  | [PSPTO_4786](https://www.ncbi.nlm.nih.gov/gene/?term=PSPTO_4786) |  |  |  |  |
|  | [PSPTO_2883](https://www.ncbi.nlm.nih.gov/gene/?term=PSPTO_2883) | PAS_4-PAS_3 |  |  |  |
|  |  |  |  |  |  |
| F8 | [PSPTO_0912](https://www.ncbi.nlm.nih.gov/gene/?term=PSPTO_0912) | 4HB_MCP_1 | 34H | EFTRF | Yes |
|  |  |  |  |  |  |
| ACF | [PSPTO_1493](https://www.ncbi.nlm.nih.gov/gene/?term=PSPTO_1493) | 4HB_MCP_1 | 40H | - | Yes |
|  |  |  |  |  |  |
| TFP | [PSPTO_5031](https://www.ncbi.nlm.nih.gov/gene/?term=PSPTO_5031) | PilJ | 40H | - | Yes |
|  |  |  |  |  |  |
| Unassigned | [PSPTO_2441](https://www.ncbi.nlm.nih.gov/gene/?term=PSPTO_2441) | (PAS_3) x 4 | 36H | - | No |

**Table S3.** Number of *Pseudomonas syringae* pv. tomato DC3000 cells with chemotaxis arrays

|  | **F6 arrays** | **F8 arrays** |
| --- | --- | --- |
| **WT** | 15 | 6 |
| **Δ*cheA1*** | 15 | 0 |
| **Δ*cheA2*** | 13 | 8 |

Supplementary information – Figures


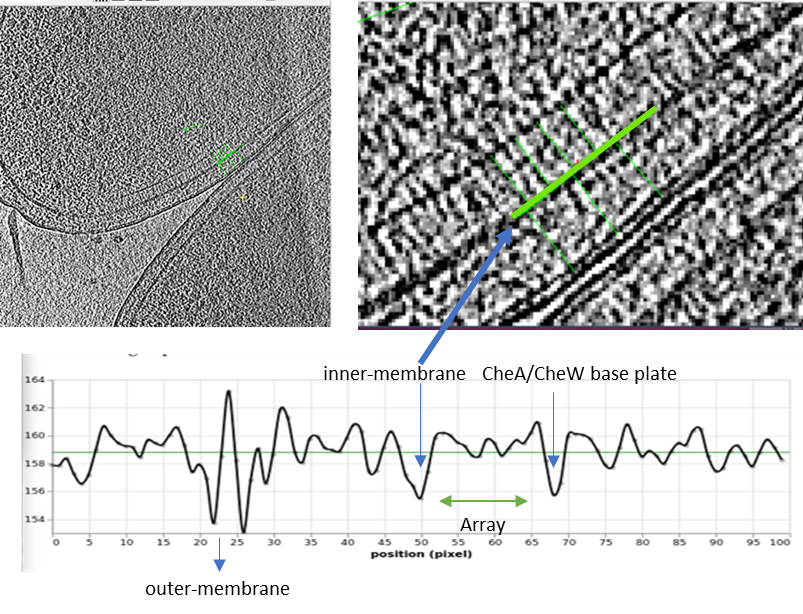


**Figure S1**. Representative average density profile of an F6 chemotaxis array in a Δ*cheA1* deletion mutant. A tomogram was used to generate model points along the inner membrane (green line) and calculate the average pixel value perpendicular to the model points. Based on the pixel values, the distance between the inner membrane and the base plate could be determined and thus the receptor height could be calculated.

**Figure S2**. Swimming motility assays of complemented strains. (A) Swimming motility assay of WT, Δ*cheA2*, and Δ*cheA2*-C strains in M9 10 mM citrate. Bars represent means of three independent experiments with four technical replicates each. Values that are significantly different are indicated by asterisks (**p* < 0.05; *****p* < 0.0001). (B) Swimming motility assay of WT, Δ*cheY2*, and Δ*cheY2*-C strains in M9 10 mM citrate. Bars represent means of three independent experiments with four technical replicates each. Values that are significantly different are indicated by asterisks (*****p* < 0.0001).

**Figure S3.** Representative image of the inner morphology displayed by colonies grown on KB supplemented with Coomassie and Congo Red. Experiments were conducted in triplicate with six technical replicates each.

**Figure S4**. Symptom development in tomato leaves by the WT, Δ*cheA3*, and Δ*wspR* strains. Bars represent the mean of the necrotic areas measured in tomato leaves. Three independent experiments were conducted with three technical replicates each.

**Figure S5**. Growth curve of WT and mutant strains in M9 minimal medium supplemented with 10 mM citrate. Measurements were performed each hour for 7 h and a final point at 24 h.
